# Longitudinal analysis of bovine mammary gland development

**DOI:** 10.1007/s10911-023-09534-0

**Published:** 2023-05-30

**Authors:** Alysia L. Vang, Tiago Bresolin, Waneska S. Frizzarini, Guilherme L. Menezes, Thiago Cunha, Guilherme J. M. Rosa, Laura L. Hernandez, Joao R. R. Dorea

**Affiliations:** 1 Department of Animal and Dairy Sciences, University of Wisconsin-Madison, Madison, WI 53706 USA; 2 Department of Animal Sciences, University of Illinois-Urbana-Champaign, IL 61801 Urbana, USA

**Keywords:** Bovine, Holstein, Mammary gland, Ultrasound, Development

## Abstract

Many studies on bovine mammary glands focus on one stage of development. Often missing in those studies are repeated measures of development from the same animals. As milk production is directly affected by amount of parenchymal tissue within the udder, understanding mammary gland growth along with visualization of its structures during development is essential. Therefore, analysis of ultrasound and histology data from the same animals would result in better understanding of mammary development over time. Thus, this research aimed to describe mammary gland development using non-invasive and invasive tools to delineate growth rate of glandular tissue responsible for potential future milk production. Mammary gland ultrasound images, biopsy samples, and blood samples were collected from 36 heifer dairy calves beginning at 10 weeks of age, and evaluated at 26, 39, and 52 weeks. Parenchyma was quantified at 10 weeks of age using ultrasound imaging and histological evaluation, and average echogenicity was utilized to quantify parenchyma at later stages of development. A significant negative correlation was detected between average echogenicity of parenchyma at 10 weeks and total adipose as a percent of histological whole tissue at 52 weeks. Additionally, a negative correlation between average daily gain at 10 and 26 weeks and maximum echogenicity at 52 weeks was present. These results suggest average daily gain and mammary gland development prior to 39 weeks of age is associated with development of the mammary gland after 39 weeks. These findings could be predictors of future milk production, however this must be further explored.

## Introduction

Bovine mammary gland development has been broadly studied, although there is limited research on longitudinal measures of development from individual animals because euthanasia of the animals is often required to collect tissue samples to examine. In addition, the period between weaning and the first lactation is often overlooked as heifers are not producing milk at this stage of development. The use of ultrasound technology could provide a noninvasive approach to monitor mammary gland development in the period between birth and the first lactation. Research on prepubertal bovine mammary gland ultrasound imaging is limited, therefore validation of this technology by other imaging methods such as histological analysis is needed.

Mammary gland growth and development is essential for future milk production and can be visualized using ultrasound technology [40]. Development of the mammary gland begins early in fetal life, but primarily occurs postnatally, particularly during the post-pubertal stage. Before puberty, much of the growth involves overall expansion of tissue mass and development of the rudimentary ductal network [22]. During puberty, the rudimentary ductal system undergoes dramatic elongation and branching [22]. Drastic changes of the mammary gland occur during pregnancy and lactation, when alveolar differentiation and maturation occurs, as well as further branching of the ductal system [22]. Although most of the secretory tissue development does not take place until pregnancy and early lactation, the period between birth and puberty is critical as it sets the foundation for pubertal ductal morphogenesis [22]. Further, nutritional effects have also been studied in post-weaned heifers although the data is conflicting. Research has suggested that high energy diets post-weaning to produce rapid weight gains to achieve puberty at earlier ages can lead to lower lifetime milk productivity through increased adiposity of the parenchyma limiting ductal expansion, although others have suggested that early breeding may be responsible for suboptimal development [10, 12, 16, 38]. Research also suggests that there are epithelial-stromal interactions during development that both support and inhibit epithelial development therefore larger fat pads may be beneficial because the fat pad dictates the extent of mammary epithelium expansion [1, 4, 11, 18, 26, 27, 31, 37]. Conflicting results in this area of research necessitate additional research to better understand mammary gland development, such as the use of repeated ultrasound measures during development.

Ultrasonography is a useful tool to visualize and diagnose various physiological and pathological conditions in animals and is already widely used by large animal veterinarians during routine herd checks and animal diagnostics and treatments. Although very few studies have utilized ultrasound to visualize and measure structures and growth within the mammary gland, there is research that has determined that ultrasound has the potential to be a valuable tool in monitoring mammary gland development [1, 2, 6, 7, 32]. Previous research determined that parenchymal area measured in ultrasound images was related to the total amount of parenchymal tissue collected, indicating that ultrasound is effective at quantification of parenchyma in heifers [1, 11]. While ultrasound has been shown to be a potentially useful noninvasive tool for evaluation of mammary development, histological findings are needed to compare with ultrasound to establish the accuracy and reliability [26]. Additionally, studies which euthanized animals for tissue collection were unable to evaluate milk production.

Therefore, the goal of this research was to study the mammary growth development using non-invasive imaging techniques and investigate the relationship of image-based features with invasive measurements obtained from histological tissue biopsies during the prepubertal phase of development in growing Holstein heifers. To this end we fed dairy calves two different milk replacers during the pre-weaning phase (first seven weeks of age) that consisted of high protein and high fat, compared to low protein and low fat to stimulate different mammary growth patterns.

## Materials and methods

### Calf management

All procedures were approved by the Animal Use and Care Committee of the University of Wisconsin – Madison (A006270-R01). The calves were born at the Blaine Dairy Cattle Center, Arlington, Wisconsin. Following a 1-week adaption period, 36 female Holstein calves (40 ± 5.42 kg) were paired by birthweight (Bw) and placed on 1 of 2 treatments intended to create mammary gland growth differences that could be detected by ultrasound.

The high (H) nutritional value diet consisted of milk replacer (Cow’s Match ColdFront Protein Blend, Land O Lakes; 27% CP, 20% Fat) fed 1 gallon twice daily and ad libitum starter grain (18% CP guaranteed analysis; UW Calf Starter – Medicated Rum/Clar,Vita Plus, Lake Mills Feed and Grain Inc., Lake Mills, WI). The low (L) nutritional value diet consisted of milk replacer (Herd Maker Protein Blend, Land O Lakes; 22% CP, 15% Fat) fed 2 quarts twice daily. Dairy calves were raised in individual calf hutches and L calves were pair-fed starter grain based on consumption by their paired H calf. The starter refusal was weighed daily, and the L calves were fed the amount of starter the H calf consumed the previous day. Calves were gradually weaned from milk replacer beginning at 6 weeks and completely weaned by 7 weeks of age. At 8 weeks of age, the calves were transitioned to ad libitum grower grain (15% CP guaranteed analysis; Vita Plus, Lake Mills Feed and Grain Inc., Lake Mills, WI). At 12 weeks of age, the heifers were moved to the Marshfield Agricultural Research Station, Stratford, WI, and transitioned from grower grain to standard total mixed ration (TMR). The animals were first transitioned to a light TMR diet for 4 to 8 weeks (45% haylage, 27.7% ground shell corn, 16.6% corn silage, 8.8% soybean meal, and 1.3% vitamins and minerals). They were then transitioned to a medium TMR diet until 12 months of age (49% haylage, 42.7% corn silage, 4.0% soybean meal, 2.8% whey, 0.8% vitamins and minerals, and 0.4% urea). At 12 months of age the animals are transitioned to the farm’s breeding diet (44.1% haylage, 32.4% corn silage, 18.1% urea 1.3% soybean meal, and 3% vitamin and minerals).

### Tissue and blood collection

Mammary gland biopsies were performed on heifers at 10, 26, 39, and 52 weeks of age. Ultrasound (Mindray Z5 Ultrasound, Mindray 65C15EA 6.5 MHz Micro-Convex Ultrasound Transducer) was used to determine the location of the biopsy. A scalpel was used to create a 1 to 2 cm slit through the skin and the capsule was dissected to allow for tissue cores to be taken with Integra Miltex disposable biopsy punches. The 2 mm punch was used at 10 weeks and the 6 mm punch was used at 26, 39, and 52 weeks of age. The tissue size varied based on age and animal. At 10 weeks of age, the tissue collected was approximately 2 mm by 5 mm. At 26 weeks of age, the tissue cores were approximately 6 mm by 1 to 2 cm, and beyond 26 weeks of age, the tissue was approximately 6 mm by 2 to 3 cm. Tissue was rinsed with saline and fixed for 24 h in 10% buffered formalin and then transferred to 70% ethanol. The tissue samples were then sent to the University of Wisconsin – Madison Veterinary School of Medicine to be embedded, sectioned, and stained with hematoxylin and eosin.

Coccygeal blood samples were collected at each biopsy as well as weekly from 8 to 14 months of age to establish cyclicity. Progesterone concentrations were determined by radioimmunoassay (MP Biomedicals).

### Image analysis

**Fig. 1:**
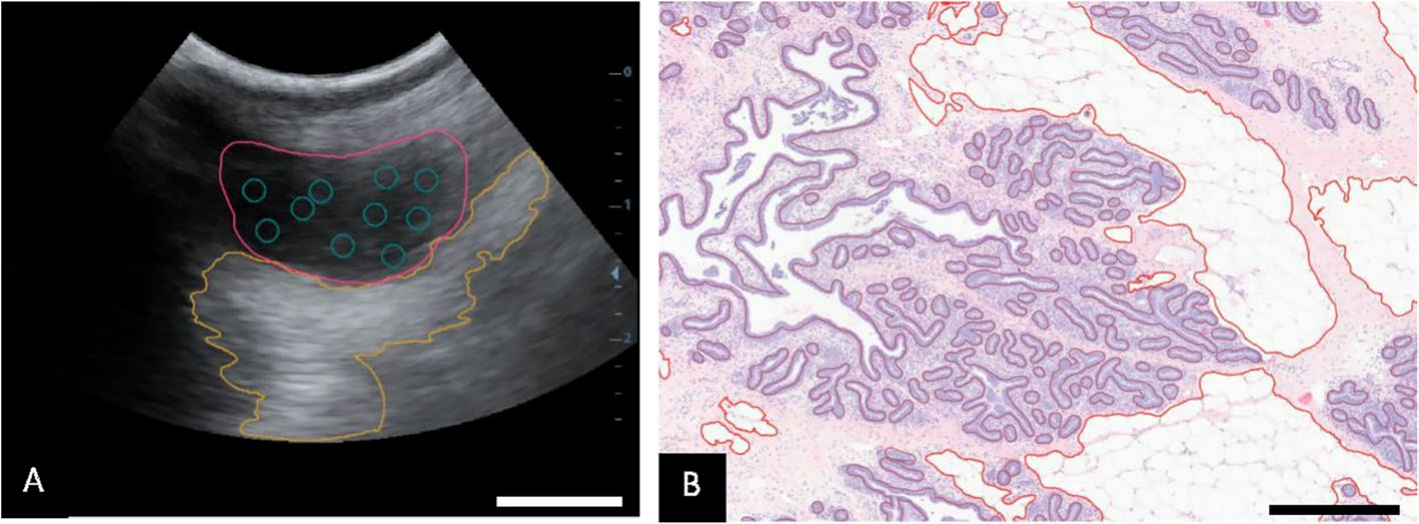
Annotation of an ultrasound image and histology image. Image A demonstrates annotation an area of parenchyma (pink), an area of fat pad (orange), and average echogenicity, which was averaged between 10 circles. Image B demonstrates annotation of the histology images in which adipose tissue is annotated in red and ductal structures are annotated in purple. The white bar in the ultrasound images measures 1 cm and the black bar in the histology images measures 500 μm

Hematoxylin and eosin-stained sections were imaged at 10 × magnification (Basler Ace 5.0 MP, Zeiss Axio Vert A1) and annotated using QuPath, an open-source software package for digital pathology image analysis [3]. In total, 72,201 objects were annotated from 132 whole-mount images. Using QuPath, the following features were annotated and calculated from whole-mount images of the histology sections: percentage of ductal tissue in the whole-mount image, average ductal area, and average maximal ductal diameter, percentage of adipose tissue in the whole-mount image (Fig. 1).

Ultrasound videos were collected prior to biopsies and frames were extracted using ffmpeg [39]. One frame from each animal at each timepoint was chosen for analysis by QuPath. Average area of parenchyma tissue and fat pad as well as average echogenicity were calculated at 10 weeks of age (Fig. 1, Table 3). Because tissue boundaries are less clear after 10 weeks average echogenicity was used to quantify parenchymal tissue density in ultrasound images at 26, 39, and 52 weeks of age. Adipose tissue appears hyperechoic or brighter, compared to parenchymal tissue which appears more hypoechoic or darker (Fig. 1). Average echogenicity was calculated by averaging the echogenicity of 10 circles placed on the image as depicted in Fig. 1.

### Statistical analyses

A linear model (R version 4.2.1, stats package version 4.2.1) including birthweight as a covariate was used to evaluate the effect of diet on area, circularity, solidity, perimeter and maximum and minimum diameter of parenchyma as well as on area, circularity, solidity, maximum and minimum diameter, mean echogenicity, echogenicity standard deviation, minimum and maximum, and perimeter of the fat pad at 10 weeks of age. Circularity measures the roundness of an object and solidity gives a measurement of the compactness of an object. A circularity value of 1 indicates a perfect circle and a perfectly convex shape has a solidity of 1. A linear mixed model (lme4 package version 1.1–30) was used to analyze weight, average duct area, average duct max diameter, total duct area / whole tissue, total adipose area / whole tissue, average echogenicity, echogenicity standard deviation, minimum and maximum echogenicity at all timepoints, which can be described as follows:

$${y}_{ijkl}=\upmu +{D}_{i}+{W}_{j}+{\left(DxW\right)}_{ij}+ {{B}_{W}}_{k}+{A}_{l}+{\mathrm{e}}_{ijkl}$$

where yijkl represents the response variable of interest, µ is the model intercept, Di is the fixed effect of the ith diet (high and low), Wj is the fixed effect of the jth week (10, 26, 39 and 52), Bw is the effect of the birthweight as a covariate, Al is the random effect of animal, and eijkl is the independent identically distributed normal error. For all models, residual analysis was performed to verify the model assumptions of normality and homogeneous variances. Pearson correlations (corrr version 0.4.4) was used to assess the relationship between average daily gain and histological and ultrasound variables at each week (e.g., 10, 26, 39 and 52).

## Results

### Diet

The two treatments did not produce significant differences for the variables of interest in both ultrasound and histological measurements (Table 2, Table 3).

### Progesterone

Average progesterone was not significantly different between the L and H groups although levels of progesterone significantly increased with age (*P* = 0.258, *P* < 0.0001; Table 1).

**Table 1:** Progesterone Measurements from 10 to 52 weeks of age

		Treatment		*P-value*s			
Variables	Nº	H	L	W	D	W*D	Bw
Progesterone concentrations	36			< 0.001	0.157	0.151	0.788
10 Weeks	36	0.00 ± 0.00	0.00 ± 0.00				
26 Weeks	36	0.0553 ± 0.0314	0.109 ± 0.102				
39 Weeks	36	0.405 ± 0.400	0.655 ± 0.541				
52 Weeks	36	5.14 ± 1.40	7.88 ± 1.23				
Age at first cycling	36	11.0 ± 0.131	11.2 ± 0.132		0.258		0.052

Mean values of progesterone and age at first cycling by diet, High (H) and Low (L), with *P*-values indicating significance of effects of week (W), diet (D), the interaction of week*diet (W*D), and birthweight (Bw) on the response variables

### Histology

**Fig. 2:**
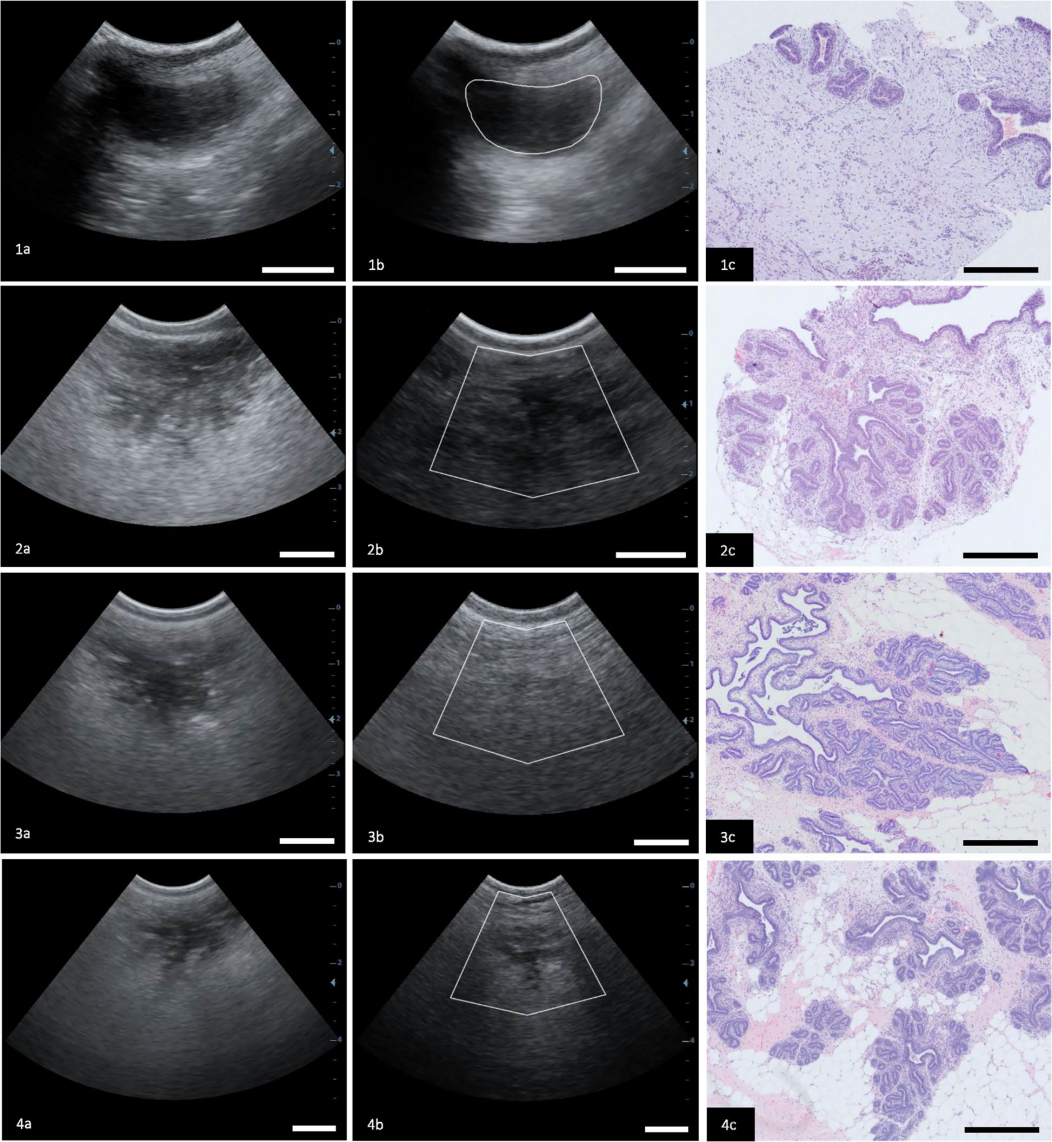
Appearance of ultrasound images near the teat displaying the development of the ductal tree (1a-4a), the approximate area of the biopsy outlined in white (1b-4b), and histology images (1c-4c) at various stages of growth. All images are from the same animal (Emily) at 10 weeks (1a-1c), 26 weeks (2a-2c), 39 weeks (3a-3c), and 52 weeks (4a-4c). The white bar in the ultrasound images measures 1 cm and the black bar in the histology images measures 500 μm. Biopsies were performed on the same 36 animals for all 4 timepoints

At 10 weeks of age, we found little adipose tissue present within the histology sections taken from the glandular portion of the mammary gland. This is supported by ultrasound images in which the parenchyma is largely separate from the fat pad (Fig. 1, Fig. 2). At 26, 39, and 52 weeks of age, the growth of ductal structures into the fat pad seen in ultrasound images is also visible in histology images, as the amount of intralobular and interlobular adipose tissue is increased in the biopsies retrieved, compared to the 10-week histology images (*P* < 0.001, Table 2). In addition, the average duct area and average duct maximum diameter decreases over time (*P* < 0.001, Table 2).

**Table 2:** Histological and Ultrasound Measurements from 10 to 52 weeks of age

		Treatment			*P-value*s			
Variables	Nº	H	L		W	D	W*D	Bw
Weight (kg)	126	258 ± 5.18	260 ± 5.26		< 0.001	0.945	0.998	0.030
Average daily gain (kg/day)	126	0.988 ± 0.046	0.854 ± 0.048		< 0.001	0.903	0.706	0.406
Average duct area (µm)	126	9.32 ± 0.066	9.16 ± 0.067		< 0.001	0.269	0.490	0.297
Average duct max diameter (µm)	124	5.04 ± 0.037	4.97 ± 0.038		< 0.001	0.446	0.672	0.195
Duct area**/**whole tissue (%)	126	8.50 ± 0.60	7.90 ± 0.60		0.156	0.760	0.946	0.266
Adipose area**/**whole tissue (%)	126	21.6 ± 1.70	23.9 ± 1.80		< 0.001	0.591	0.914	0.183
PAR average echogenicity	126	0.240 ± 0.009	0.219 ± 0.010		0.308	0.675	0.792	0.401
PAR echogenicity SD	126	0.040 ± 0.002	0.041 ± 0.002		0.886	0.155	0.069	0.003
PAR echogenicity min	126	0.102 ± 0.007	0.099 ± 0.008		0.303	0.751	0.613	0.853
PAR echogenicity max	126	0.447 ± 0.014	0.415 ± 0.014		0.101	0.480	0.833	0.515

Mean values of histological and ultrasound variables of interest listed by diet, High (H) and Low (L), with *P*-values indicating significance of effects of week (W), diet (D), the interaction of week*diet (W*D), and birthweight (Bw) on the response variables. Echogenic values range from 0 (white) to 1 (black)

### Ultrasound

**Fig. 3:**
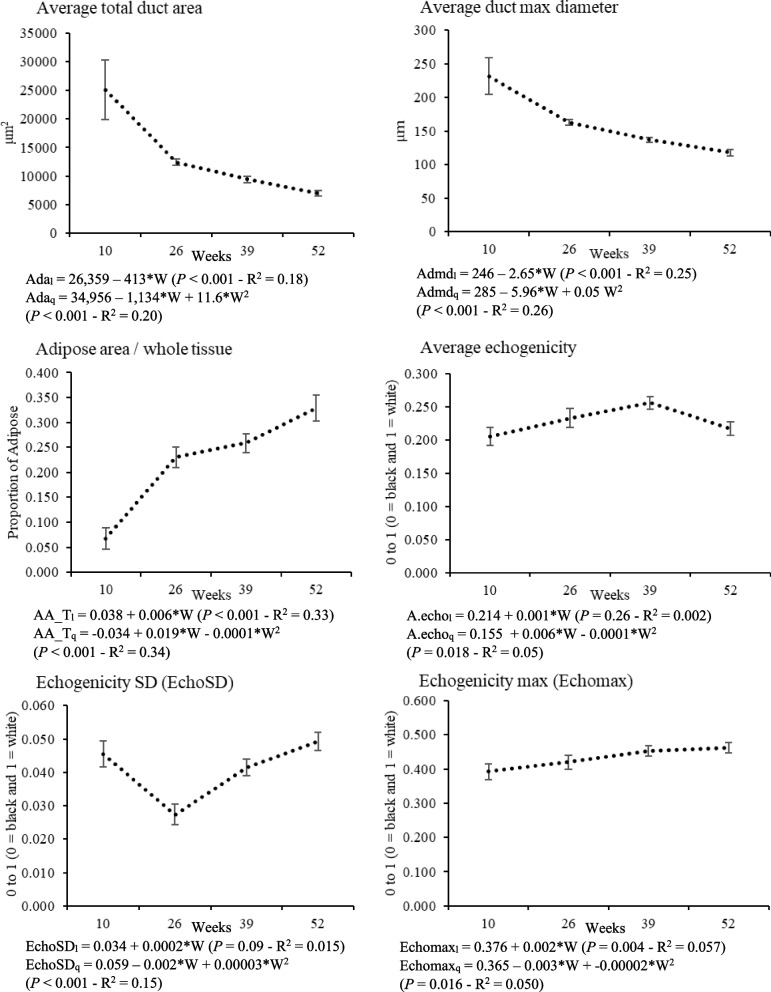
Means of histological and ultrasound variables with a significant effect (*P* < 0.05) in relation to week. Echogenic values range between 0 and 1 (0 = black and 1 = white). l = linear effect, q = quadratic effect, and W = weeks

Changes in mammary gland morphology can be seen in ultrasound and histology images over time (Fig. 2). Echogenicity values ranged from 0 to 1, with 0 representing white and indicating adipose tissue and 1 representing black indicating parenchymal tissue. At ten weeks of age, the parenchyma is largely contained and appears as a round hypoechoic region, indicating a solid mass of dense tissue. The fat pad is present as a hyperechoic region below the parenchyma. There was no significant difference in parenchymal area or visible fat pad area between the two treatment groups (*P* = 0.980, 0.633; Table 3). Around 10–12 weeks of age, ductal structures are seen emerging from the parenchymal region and into the fat pad. At 26 weeks, the defined edges of the mammary gland are ambiguous in ultrasound although ductal growth is clearly visible through 39 and 52 weeks. Due to the ambiguity of boundaries, mean echogenicity was utilized to quantify parenchyma at 26, 39, and 52 weeks of age. Mean echogenicity was not significantly different between the two treatments at 10 weeks of age (*P* = 0.875, Table 3) and did not significantly change from 10 to 52 weeks of age (*P* = 0.308, Table 2, Fig. 3).

**Table 3:** 10-Week Ultrasound Measurements

		Treatment		*P-value*s
Variables	Nº	H	L	D
PAR area (cm^2^)	27	1.18 ± 0.125	1.19 ± 0.130	0.980
PAR circularity	26	0.564 ± 0.027	0.633 ± 0.027	0.056
PAR solidity	27	0.854 ± 0.016	0.892 ± 0.016	0.091
PAR min diameter (cm)	27	0.893 ± 0.00057	0.825 ± 0.0772	0.579
PAR max diameter (cm)	27	1.87 ± 0.00120	1.94 ± 0.162	0.744
PAR perimeter (cm)	27	5.13 ± 0.00265	4.80 ± 0.356	0.488
PAR echogenicity mean	27	0.207 ± 0.021	0.203 ± 0.022	0.875
PAR echogenicity SD	27	0.047 ± 0.006	0.044 ± 0.006	0.823
PAR echogenicity min	27	0.072 ± 0.016	0.083 ± 0.017	0.608
PAR echogenicity max	27	0.400 ± 0.034	0.372 ± 0.036	0.529
FP area (cm^2^)	27	2.08 ± 0.324	1.87 ± 0.339	0.633
FP circularity	27	0.525 ± 0.044	0.535 ± 0.045	0.866
FP solidity	26	0.835 ± 0.022	0.816 ± 0.023	0.521
FP min diameter (cm)	27	1.21 ± 0.000987	1.18 ± 0.136	0.898
FP max diameter (cm)	27	2.53 ± 0.0014	2.44 ± 0.201	0.733
FP perimeter (cm)	27	8.21 ± 0.00711	6.68 ± 0.962	0.232
FP echo mean	26	0.590 ± 0.021	0.622 ± 0.022	0.272
FP echogenicity SD	26	0.105 ± 0.004	0.107 ± 0.004	0.747
FP echogenicity min	26	0.217 ± 0.027	0.199 ± 0.028	0.622
FP echogenicity max	26	0.855 ± 0.016	0.875 ± 0.016	0.358

Measurements of ultrasound variables of interest at 10 weeks of age compared between diet (D) treatments, High (H) and Low (L), with corresponding *P*-values. Echogenic values range from 0 (white) to 1 (black). PAR indicates parenchyma and FP indicates fat pad. A circularity value of 1 indicates a perfect circle and a perfectly convex shape has a solidity of 1

### Correlations between ultrasound and histological features

**Fig. 4:**
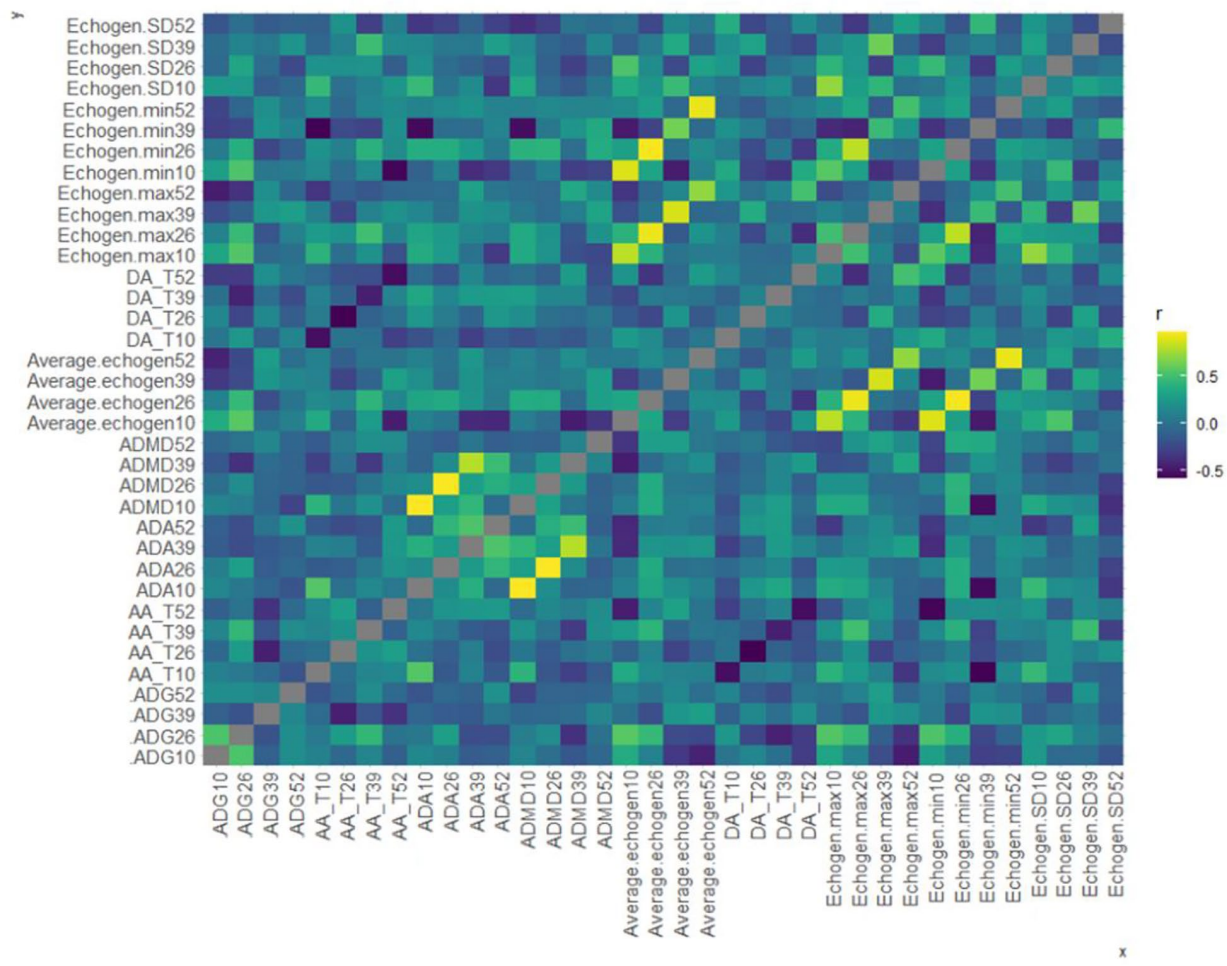
Pearson’s correlation matrix comparing ultrasound and histological variables with average daily gain (ADG). Abbreviations are as follows: adipose area / whole tissue area (AA_T), average duct area (ADA), average duct max diameter (ADMD), average echogenicity (Average echogen), duct area / whole tissue area (DA_T), maximum echogenicity (Echogen. Max), minimum echogenicity (Echogen. Min), echogenicity standard deviation (Echogen. SD), and age of the animals in weeks (10, 26, 39, 52)

There is a significant negative correlation between average echogenicity of the parenchyma at 10 weeks of age and total adipose as a percent of histological whole tissue at 52 weeks of age (*r* = -0.458, *P* = 0.037; Fig. 4). In addition, there is a strong negative correlation between average daily gain at 10 weeks and maximum echogenicity of the parenchyma at 52 weeks (*r* = -0.465, *P* = 0.004, Fig. 4). Similarly, there is a negative correlation between average daily gain at 26 weeks and maximum echogenicity of the parenchyma at 52 weeks (*r* = -0.367, *P* = 0.0027, Fig. 4).

## Discussion

The objective of this study was to investigate the growth development of mammary gland tissue of growing calves and study the associations between image-based features obtained from mammary gland ultrasound images during the prepubertal phase of development in Holstein heifers with histological analysis acquired through tissue biopsy. Several studies have demonstrated that ultrasound images can be an effective tool to quantify specific tissues (e.g., parenchyma) in the mammary gland [2, 7, 11, 13, 17, 19, 20, 24, 32, 33]. These studies have demonstrated that ultrasound images may also generate relevant qualitative features of these tissues, such as parenchymal area in young heifers as well as echogenicity, which can be used as an indicator of tissue composition (e.g., protein, fat).

In 10-week-old heifers, the mammary gland appeared as an oval to fusiform hypoechoic area largely separate from the fat pad. This changes dramatically by 26 weeks of age as ducts are extending into the fat pad (Fig. 2). This ductal development continues through 39 and 52 weeks of age. As the ductal structures expand outward into the fat pad, the overall echogenicity of the mammary gland would increase due to the higher echogenic value of adipose tissue. Although a difference in average echogenicity was not observed between treatments and between timepoints, the maximum echogenic value did increase from 0.392 at 10 weeks of age to 0.463 at 52 weeks of age. The increase in max echogenicity over time in both groups is consistent with the increase in adipose tissue as well as the decrease in the percentage ductal area seen in histology images (Fig. 2). Overall, the ductal area as a percentage of the whole tissue did not significantly change over time, ranging from 6.8% to 9.2%, suggesting that with the increase in mammary gland size, there is also an increase in ductal numbers, as the average ductal area and average maximum ductal diameter decrease over time. This increase in ductal numbers can also be visualized in the histology images shown in Fig. 2. It is presently unclear how these structural changes within the mammary gland influence future milk production.

There is an interesting significant negative correlation between average echogenicity of the parenchyma at 10 weeks of age and total adipose as a percent of whole tissue at 52 weeks of age (*r* = -0.458, *P* = 0.037; Fig. 4). This suggests that higher values of parenchymal echogenicity at 10 weeks of age is correlated with less overall adipose tissue at 12 months of age. In addition, there are strong negative correlations between average daily gain at 10 weeks as well as average daily gain at 26 weeks and maximum echogenicity at 52 weeks (*r* = -0.465, *r* = -0.367; *P* = 0.004, 0.0027; Fig. 4). This could suggest that average daily gain prior to 39 weeks of age is associated with the development of the mammary gland after 39 weeks of age and these effects could have long-lasting impacts on future milk production, although it is unclear what those impacts may be. Higher parenchymal echogenicity at 10 weeks may be due to earlier growth of the ductal structures into the mammary fat pad because ductal structures tend to have lower echogenicity therefore any ductal growth into the mammary fat pad would increase parenchymal echogenicity and decrease overall echogenicity of the fat pad. Additional research is required to investigate the long-term effects of early development and whether ultrasound is a reliable method for predicting future lactation performance.

The presented data associations suggest that animals with higher body growth rates early on in life may have better utilized dietary nutrients and changed the rate and the timing of ductal growth into the mammary fat pad. In our experiment, the grain intake was not fully restricted to low calves due to animal welfare concerns combined with a large sample size (200 calves total, 36 biopsied) and extreme winter period. Limiting grain intake in low nutritional plan may not have been enough to dramatically limit tissue development. Although we did not find effect of nutritional strategies on mammary tissue, the association with ADG regardless the dietary effect may suggest such pattern. Other studies have showed dramatic increased mammary gland growth due to enhanced diets preweaning and stunted mammary development with high energy diets post-weaning [15, 34]. One study found that calves fed milk replacer with higher protein and fat content had increased estrogen receptor 1 expression intensity [15]. Because the estrogen receptor is involved in insulin-like growth hormone signaling, it is thought that enhanced feeding primes the mammary gland to better respond to mammogenic hormone stimulation [15, 28]. The molecular and hormonal interactions involved in mammary gland growth are complex and not yet fully understood, although these results suggest that mammary gland growth and development is highly responsive to nutrition, therefore additional research is required to better understand the long-term effects of stunted or enhanced growth.

Mammary development evaluation by the noninvasive method of ultrasound could complement current methods of replacement heifer selection as well as provide researchers an alternative to culling of animals to assess tissue development. The image-based features extracted from the mammary gland tissue could be used in combination to a series of variables such as genomics, weather, body growth development, and diet to predict the lactation potential of dairy cattle. Building management tools for early decision is critical to create profitable and sustainable dairy production systems. The economic and environmental costs to raise a heifer that will not be an efficient cow due to lack of glandular tissue could be avoided if phenotyping technologies can generate large-scale and precise animal measurements. Besides, there is a tremendous value for these type of phenotypes for genetic selection in livestock animals. Although genomic prediction can rank animals based on lactation potential, environmental effects play a critical role on future performance as unexpected events such as health issues, heat-stress, suboptimal nutrition may negatively affect the performance of animals, including the ones with high genetic potential [9, 14–16, 35, 36].

The development of non-invasive tools to evaluate glandular tissue may also contribute with lactation monitoring in humans. Human and bovine mammary gland development and microstructure are quite similar, and ultrasound has been used to evaluate breast maturation at puberty with success, indicating that the sensitivity of ultrasound enables the visualization of changes consistent with the start of pubertal breast development that prior evaluation methods such as palpitation or staging did not pick up [5, 20, 30]. There has been limited research on human breast development and lactation potential and so the development of ultrasound as a method to evaluate glandular tissue may allow for the development of much needed evidence-based interventions and development of postpartum plans for individuals prior to parturition to avoid common issues associated with low milk production. Ultrasound is typically used to diagnose breast disorders such as growths, abscesses, and lesions in lactating and nonlactating breasts and can be very effective in identifying abnormalities [20, 21, 23, 25, 29]. Few studies have utilized ultrasound to image and investigate the structure and function of lactating human breasts. One study did find that there was no relationship between milk production and the proportion of glandular, proportion of adipose tissue, or the size of ducts [30]. Although the mentioned study is a great reference and a starting point for further research, the sample size was small and did not follow individuals’ pre-pregnancy or pre-partum through the lactation. Some individuals cannot breastfeed due to a lack of glandular tissue or experience delayed onset of lactation for any number of reasons including stress, malnutrition, genetics, and other environmental effects. Although there was no relationship between milk production and the proportion of glandular tissue and adipose tissue, further investigation is required to completely understand milk production and the complex mechanism of lactation in humans [8]. In addition, time points representing the age beyond the data collected in our study are necessary to understand when mammary tissue development stabilize for variables that presented linear trend. Longer longitudinal studies following individuals from pre-pregnancy or prepartum through the end of lactation could provide insight into lactation success or failure and the factors that affect lactation even before it begins. Thus, a study as ours can serve as a model to study lactation potential through the use of non-invasive imaging tools.

## Conclusions

In the present study, nutritional strategies implemented early on in life did not alter the mammary gland development measured by ultrasound and histological images from 10 to 52 weeks of age. Our results did not agree with most of the published literature, where low nutritional plans were more restrictive. Image-based features obtained from ultrasound images can be used to assess mammary gland development, and these features are associated with tissue development measured through histological images. Such finding will allow for the development of predictive analytics using ultrasound images as potential inputs to evaluate the development of mammary gland fat pad and ducts. Additional studies should be performed to evaluate when the tissue development reaches the plateau for variables that presented a linear effect in relation to weeks of age, and to increase the image dataset for future validation of ultrasound image features as predictors of parameters obtained from tissue biopsies, as histological images. The 36 animals followed in this study will continue to be followed through their first lactation. With additional data from these animals it may be possible to determine whether our findings could be predictors of future milk production.

## Data Availability

The datasets generated during and/or analyzed during the current study are available from the corresponding author on reasonable request.
